# Thousands of exon skipping events differentiate among splicing patterns in sixteen human tissues

**DOI:** 10.12688/f1000research.2-188.v2

**Published:** 2013-11-21

**Authors:** Liliana Florea, Li Song, Steven L Salzberg

**Affiliations:** 1 Center for Computational Biology, McKusick-Nathans Institute of Genetic Medicine, Johns Hopkins University School of Medicine, Baltimore, MD, 21205, USA; 2 Department of Medicine, Johns Hopkins University School of Medicine, Baltimore, MD, 21205, USA; 3 Department of Computer Science, Johns Hopkins University, Baltimore, MD, 21205, USA; 4 Department of Biostatistics, Bloomberg School of Public Health, Johns Hopkins University, Baltimore, MD, 21205, USA

## Abstract

Alternative splicing is widely recognized for its roles in regulating genes and creating gene diversity. However, despite many efforts, the repertoire of gene splicing variation is still incompletely characterized, even in humans. Here we describe a new computational system, ASprofile, and its application to RNA-seq data from Illumina’s Human Body Map project (>2.5 billion reads).  Using the system, we identified putative alternative splicing events in 16 different human tissues, which provide a dynamic picture of splicing variation across the tissues. We detected 26,989 potential exon skipping events representing differences in splicing patterns among the tissues. A large proportion of the events (>60%) were novel, involving new exons (~3000), new introns (~16000), or both. When tracing these events across the sixteen tissues, only a small number (4-7%) appeared to be differentially expressed (‘switched’) between two tissues, while 30-45% showed little variation, and the remaining 50-65% were not present in one or both tissues compared.  Novel exon skipping events appeared to be slightly less variable than known events, but were more tissue-specific. Our study represents the first effort to build a comprehensive catalog of alternative splicing in normal human tissues from RNA-seq data, while providing insights into the role of alternative splicing in shaping tissue transcriptome differences. The catalog of events and the ASprofile software are freely available from the Zenodo repository

( http://zenodo.org/record/7068; doi: 10.5281/zenodo.7068) and from our web site http://ccb.jhu.edu/software/ASprofile.

## Background

Alternative splicing is a widespread phenomenon in eukaryotic species, and differential regulation of alternative splice variants is gaining recognition as an important mechanism of gene regulation. More than 90% of human genes are estimated to be alternatively spliced ^1, 2^, producing multiple transcripts and (often) different protein sequences from a single locus. The number of variants of a gene ranges from two to potentially thousands ^3^. The resulting proteins may exhibit different and sometimes antagonistic functional and structural properties ^4^, and may inhabit the same cell with the resulting phenotype representing a balance between their expression levels ^5^. Defects in splicing have been implicated in human diseases, including cancer ^6– 9^. Developing a comprehensive catalog of splice variant annotations across a wide range of tissues and conditions is important not only as part of our efforts to create a complete gene list for the human genome, but also to serve as a reference for differential expression studies aiming to identify molecular markers of disease.

Annotation of alternative splicing has traditionally been based on cDNA (expressed sequence tags (EST), mRNA) sequence data from public repositories such as dbEST, RefSeq ^10^, and the Mammalian Gene Collection ^11^. These data sources were compiled over many years, from independent contributions by thousands of investigators working on different genes and systems, and are therefore inconsistent in their coverage of the transcriptome in general and of each gene individually. Because these resources were generated using Sanger sequencing, they were relatively expensive to produce, but despite the cost have insufficient depth to capture the diversity of splicing variations in human cells. RNA-seq technology produces vastly more sequence data in a cost-effective way and in a much shorter amount of time, allowing a deep characterization of the transcriptome in a variety of cells and conditions ^2, 12, 13^, but so far little has been done to systematically assess its potential ^14^. Starting from one of the most complete sets of RNA-seq data available, the Illumina Human Body Map, we addressed the questions: "how much alternative splicing do we find?" and "how does alternative splicing vary among tissues?" We used this data set, spanning 16 tissues and containing over 2.5 billion sequences, to build a comprehensive catalog of alternative splicing (AS) within each tissue. We also compared AS profiles across tissue types to derive insights into the role of AS in shaping transcriptome differences.

## Results

We analyzed the Illumina Human Body Map RNA-seq set (ArrayExpress accession: E-MTAB-513; http://www.ebi.ac.uk/arrayexpress), consisting of approximately 160 million reads from each of 16 tissues, each from a different individual. This resource is one of the most high-quality and complete to date, and therefore allows us to detect AS events with high accuracy. To determine splicing variations in each tissue, we first mapped reads to the reference genome and assembled them into transcripts or transcript fragments. We then analyzed the transcripts to determine putative alternative splicing events, in particular exon skipping events, within and between samples, and compared them across the tissues. We focused on exon skipping because the alignment evidence for these events is usually clear and unambiguous, and less likely to be confounded by alignment or assembly artifacts. The data support a number of overall findings:

1. Based on a comparison against several annotation databases (Ensembl ^15^, CCDS ^16^, UCSC Genes ^17^ and H-ASDB ^18^), we found that 11–45% of the assembled transcripts in each tissue were unannotated, as well as a majority (65%) of the 26,989 exon skipping events discovered from this data set.

2. These novel events appear to be more tissue-specific than previously annotated (known) events; i.e., they tend to occur in fewer tissue types.

3. When an exon is skipped, it usually occurs in a different tissue from those in which it is present; only 5–23% of events express both forms within the same tissue.

4. Comparing exon skipping profiles across tissues, we found that only 10–20% of the events identified show different splicing ratios between any two given tissues, whereas 50–65% of the cataloged events are not present in either or both tissues.

Overall, our analysis reveals a complex and dynamic picture of alternative splicing across tissue types, where differences among tissue transcriptomes arise from the interplay between constitutive transcription and alternative splicing. Most importantly, we compiled the first large repository of putative exon skipping and other classes of alternative splicing events in normal human tissues detected from RNA-seq data, which will be a valuable resource for studies of regulation and to identify markers of diseases. This catalog and our methods, implemented in the open source software program ASprofile, are freely available under the GNU GPL license from the Zenodo repository ( http://zenodo.org/record/7068; doi:10.5281/zenodo.7068) and from our web site http://ccb.jhu.edu/software/ASprofile.

### A global view of alternative splicing in the 16 tissues

To determine alternative splicing events and globally characterize alternative splicing within a given tissue, we analyzed 50-bp paired-end sequences from 16 different tissues: adrenal, adipose, brain, breast, colon, heart, kidney, liver, lung, lymph, ovary, prostate, skeletal muscle, testes, thyroid and white blood cells. These data are publicly available as the Illumina Human Body Map project (EMBL accession ENA-ERP000546; ArrayExpress accession: E-MTAB-513; http://www.ebi.ac.uk/arrayexpress/browse.html?keywords=E-MTAB-513&expandefo=on). Libraries were made from polyA-selected mRNA with an insert size of 210 bp, independently for each tissue, using a random priming process and unstranded. One run of 2x50 bp paired-end sequencing was performed on the Illumina HiSeq2000 instrument, using one lane per tissue, to produce approximately 80 million pairs of reads (160 million sequences) per tissue. The entire data set comprises ~128 gigabases (GB) of sequence (~8 GB sequence per tissue), making this one of the most complete RNA-seq resources to date and one of very few spanning multiple types of tissues.

**Figure 1.**
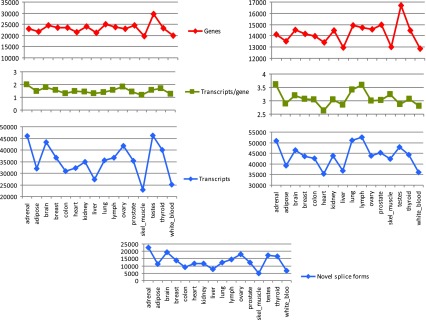
A high-level view of alternative splicing in sixteen human tissues: numbers of multi-exon ‘genes’ and transcripts from *de novo* transcript assemblies produced by Cufflinks (left), and by Cuffcompare (right). Since Cufflinks may break transcripts and genes into multiple fragments when there is insufficient read coverage, we used Cuffcompare to compare transfrags against the Ensembl reference annotations to produce a better estimate for the number of genes and transcripts in the samples. Results in the right panel show the total number of Ensembl annotated as well as novel genes, and respectively transcripts, found in each sample. The number of novel isoforms identified by Cuffcompare is shown in the bottom panel.

We mapped reads to the human genome with the program TopHat ^19^ ( Supplemental Table S1), and then assembled overlapping reads on the genome into transcript fragments using Cufflinks ^20^, which showed the best accuracy in testing ( Supplemental Table S2). Cufflinks represents all reads at a locus as an assembly graph, in which any two reads are connected if they overlap and have compatible splice patterns, and then traverses the graph to produce the minimum number of transcripts that can explain all of the input reads. Because single-exon transcripts, which form the bulk of the assemblies ( Figure 1 and Supplemental Table S3), are frequently artifacts of sequencing and mapping, we used only the multi-exon transcripts to measure the gene and transcript content.

**Figure S4.**
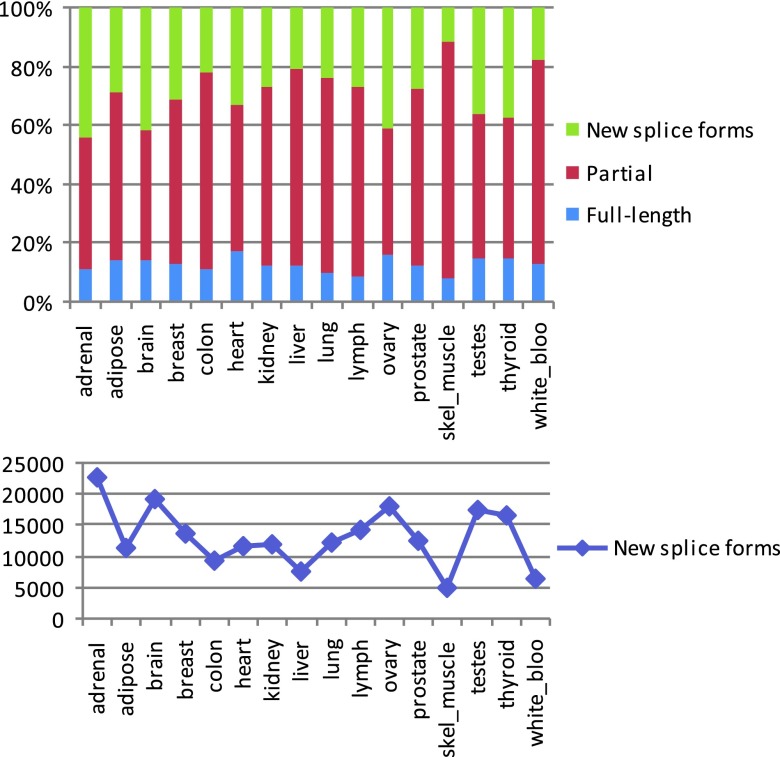
Proportion of predicted novel splice forms from the total number of transcript assemblies, including novel and known (top), and absolute counts of novel splice forms (bottom) detected in the 16 tissues. Novel and known isoforms are determined by comparison with ENSEMBL v.61 gene annotations using the program Cuffcompare (see caption to Table S4 for more details).

Although the assemblies produced by Cufflinks can be full-length transcripts, many transcripts can only be assembled into partial fragments (e.g., when the coverage of a transcript contains gaps). We therefore designate all transcript assemblies, complete or otherwise, as *transfrags*. For each tissue, Cufflinks produced between 23,000–46,000 multi-exon transfrags, clustered into 20,000–30,000 loci. The number of transfrags was greatest in brain and testes, and lowest in liver, colon, white blood cells and skeletal muscle, reflecting the combined effects of the number of expressed genes (Pearson’s r ^2^=0.74) and splicing variation within genes. These findings are consistent with some of the earlier estimates of the sizes of transcriptomes of different tissues ^1, 21– 25^. To estimate the number of novel splice forms, we compared the assemblies to a known annotation database, Ensembl ^15^, using the program Cuffcompare from the Cufflinks package. This gave us between 35,000–52,000 transfrags per tissue that were associated with 13,000–17,000 Ensembl genes, of which a large fraction (between 5,000–20,000 per tissue, representing 11–45% of the total) appeared to be novel splice forms ( Figure 1, Supplemental Figure S4 and Supplemental Table S4). Tissues with large numbers of new splice forms also had a larger fraction of candidate new splice forms.

Even with the best data and software, computational reconstruction of long transcripts from short reads is prone to assembly errors. We therefore focused on classes of alternative splicing events that are most likely to be assembled correctly. Exon skipping events are the most prevalent type of alternative splicing events in the human genome ^26^, and are particularly easy to identify from transcript data and less likely to be mis-assembled. They have been extensively studied and are well represented in the databases. For these reasons, exon skipping events provide an excellent proxy for the number of other types of splicing variants in a sample.

We define an exon skipping ‘event’ as a pairing between an exon-containing form (‘on’) and an exon-excluding form (‘off’), occurring at the same exon and with the same flanking introns. The same exon (or intron) may be involved in multiple exon skipping events, though the number of such cases is small. To generate a catalog of events for the sixteen tissues, we analyzed transcript assemblies using our software ASprofile and identified differences in exon-intron structures characteristic of the various classes.

We found over 150,000 candidate alternative splicing events ( Supplemental Table S5). Among these, we found 26,989 exon skipping events at 25,017 distinct exons, involving 22,145 distinct introns. Almost all of these events (25,920) were found in comparisons between different tissues, although a significant fraction (16,382) were also found when comparing isoforms within the same tissue. There were 1,069 instances of alternative splicing events that were restricted to a single tissue, most of them in testes (416) and brain (172).

**Figure S6.**
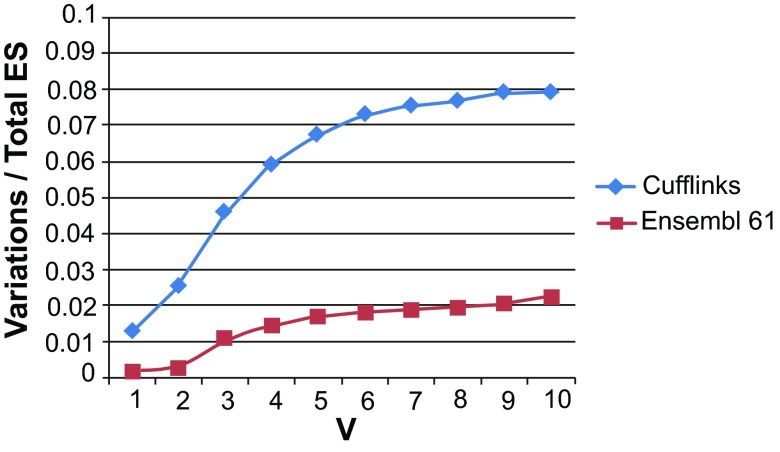
Number of event variations due to small differences in exon and intron boundaries as a fraction of the total number of events, with varying cutoffs for the allowable difference (V bp). Values shown are for exon skipping (ES) events derived from the Illumina Human Body Map data (‘Cufflinks’) and from Ensembl v.61 gene annotations, from columns 4 and 8 in Table S6.

**Figure S8.**
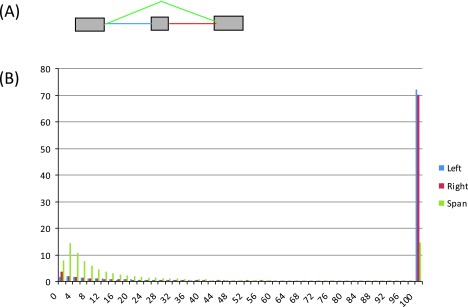
Histograms of reads in the 16 tissue samples supporting the two introns flanking the alternatively spliced exon (blue, red) and the intron spanning the exon (green), respectively, for the 26,989 identified exon skipping events. ( **A**) Diagram of an exon skipping event, illustrating the three types of introns: left – blue, right – red, and intron spanning – green. ( **B**) Read histograms for the three categories of introns. The x-axis represents the number of supporting reads for an intron, grouped in bins, and the y-axis shows the percentage of introns by levels of supporting reads (in bins). Most events have deep support (>100 reads) for the flanking introns, and to a lesser extent for the spanning intron.

Mapping artifacts can create false exon skipping events, due to incorrect or duplicated splice junctions or incorrectly reconstructed exons. To assess the accuracy of the data set and identify potential artifacts for future curation, we first looked for co-located events that showed small variations (≤5 bp) at exon and intron boundaries, which could be caused by imprecise mapping of spliced reads. Such variations could lead to redundancy in reporting the events. For reference, we compared the extent of variation against the ENSEMBL gene annotations. There were 1,822 (6.75%) events in our data set that represented slight variations of other events compared to 427 (1.7%) in the ENSEMBL data, suggesting that up to 5% of events in our data set may be redundant ( Supplemental Figure S6 and Supplemental Table S6). However, this figure is likely an overestimate, given that small 5´ and 3´ exon splicing variations are hard to detect with conventional (Sanger) data and are likely underrepresented in the reference gene annotations. We also evaluated the reproducibility of our results when using other transcript assembly methods (IsoCEM ^27^, SLIDE ^28^, and Scripture ^29^), in a second test. We found that 84% (2,471 out of 2,934) of exon skipping events found in the adrenal sample alone were independently discovered when using one of the other transcript assembly methods ( Supplemental Table S7). When aggregating data across the sixteen tissues, 92% (24,936) of the introns spanning skipped exons have at least two reads supporting them in the sixteen tissues; although in general exon-skipping introns have fewer supporting reads than other introns ( Supplemental Figure S8). Similarly, in 21,469 (80%) of the exon skipping events, the exon was present in two or more tissues. Thus, while some assembly artifacts could still be present, most of the events discovered have strong supporting evidence.

### How much alternative splicing is novel?

Simply counting the number of transcripts assembled from RNA-seq data is one way to measure the extent of alternative splicing. However, this can be confounded by transcripts that are assembled incompletely or incorrectly. Exon skipping events are discrete and easily counted, although it is worth noting that a given exon might be skipped in multiple distinct transcripts. To avoid the difficulties of counting all splice variants, we used the number of exon skipping events as a surrogate measure of splicing variation.

To identify which of the splice variants were previously unreported, we searched the 26,989 skipped exon events against four gene annotations databases: CCDS ^16^ (23,353 sequences) ( http://genome.ucsc.edu, download May 2011), UCSC Genes ^17^ (73,671) ( http://genome.ucsc.edu, download May 2011), Ensembl v.61 (120,122) ( http://ensembl.org), and H-DBAS ^18^ (58,609 mRNA and 37,096 fl-cDNA RASVs) ( http://h-invitational.jp/h-dbas/, download May 2011). Importantly, these specific data sets and releases had been produced using almost exclusively traditional cDNA (EST, mRNA) resources, and therefore provide a fairly accurate assessment of the potential to discover novel alternative splicing variation in RNA-seq experiments. We found that over 60% (17,442) of the events were novel, even after allowing for slight differences in the annotation of exon boundaries present in the various databases ( Supplemental Table S9). New exons, new introns, or both can lead to novel splicing events, but we discovered novel introns much more frequently than novel exons (2,914 exons and 15,958 introns). The majority of novel exons overlap known exons; i.e., one or both exon boundaries are novel, but not the entire exon. 884 exons did not overlap any previously annotated exon. A total of 996 (34.2%) of the novel exons and 3,801 (23.8%) of the novel introns were also supported by EST alignments, which provides independent cDNA evidence for those events.

**Figure 2.**
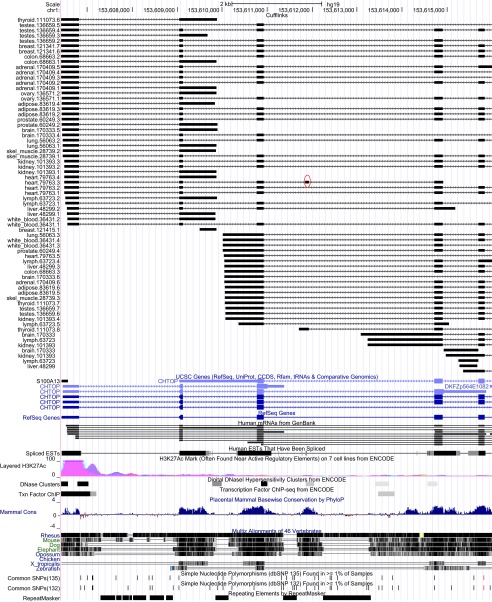
A novel alternatively spliced exon (chr1: 153,611,844–153,611,927) at the *CHTOP* gene locus, which does not overlap any known annotation. This novel 84-bp exon, marked with a red circle in the figure, is the 4 ^th^ exon in one of the transcripts from heart tissue (heart.79763.3), and it appears exclusively in that tissue, although a partial form is present in a skeletal muscle transcript. The two introns flanking the event and the spanning intron are supported by 5, 59 and 702 reads, respectively, in the 16 tissues.

One example of a novel event is shown in Figure 2. CHTOP (Chromatin target of Prmt1, synonym FOP) is a small nuclear protein on chromosome 1 characterized by an arginine- and glycine-rich region. It has a role in ligand-dependent activation repression of an estrogen receptor target gene ^30^, and has been shown to be a critical modulator of gamma-globin gene expression ^31^. The 84 bp in-frame exon at chromosome 1 positions 153,611,844–153,611,927, which we observed only in heart tissue, does not overlap any of the annotated structures for this gene and has only weak EST evidence, in the terminal exon of EST DB270513. However, the entire exonic region is highly conserved in placental mammals, strongly suggesting that this region is part of the spliced gene. Further, DNaseI hypersensitive sites lend support to an alternative transcript starting at this exon in thyroid tissue. This alternative transcription start site was also identified by our method, and is also missing from the annotation.

**Figure S10.**
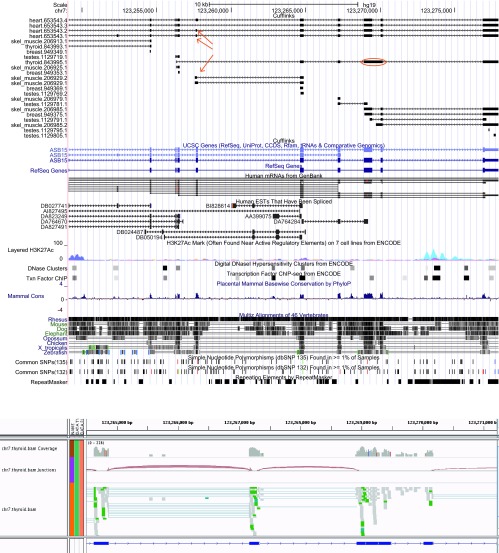
Novel exon skipping event at the human *ASB15* gene locus. **(Top)** Exon chr7:123,257,633–123,257,718, present in heart (heart.653543.1 and heart.653543.2) and with partial support in skeletal muscle tissue (skel_muscle.206929.2), is novel. Red arrows point to the exon appearing in two isoforms reconstructed in the heart sample, and to the (potentially) partial form in a skeletal muscle transcript for the *ASB15* gene. The spanning intron in the heart sample is also novel. Additionally, we found a potentially retained intron (chr7:123,269,489–123,270,019; thyroid.843995.1), circled in red, whose 531 bp sequence is conserved across multiple vertebrates. **(Bottom)** Read support for the putative intron retention event above in the thyroid sample, whereas the flanking introns are devoid of intronic reads. RefSeq exon annotations are shown in blue.

Another novel event occurs in the gene ASB15 (ankyrin repeat and SOCS box containing 15) ( Supplemental Figure S10). Human ASB15 is known to be expressed predominantly in skeletal muscle and to participate in the regulation of protein turnover and muscle cell development by stimulating protein synthesis and regulating differentiation of muscle cells. Bovine ASB15 mRNA was also found in heart and pituitary gland tissue, and rat ASB15 was additionally present in kidney and lung tissue, but the amount in most other tissues analyzed was scarce ^32^. These results are consistent with the Illumina Human Body Map data set. Here, exon chr7:123,257,633–123,257,718 is a novel shorter variant that shares its 5´ end with the annotated exon. Both the exon-containing and the exon skipping form are expressed in heart, and have strong read support in the 16 tissues (136 and 39 reads supporting the flanking introns, and 30 reads spanning the exon). Evidence for the novel splice junction is also present in skeletal muscle tissue. We also found a novel putative intron retention event (chr7:123269489–123270019, 531 bp) whose sequence is conserved across multiple vertebrate species. Overall, our analyses underscore the vast potential for RNA-seq experiments to unearth novel splicing events and isoforms.

### Characterization of exon skipping events

We next sought to characterize the set of exon skipping events within and across the sixteen tissues, which also offers a glimpse into the dynamics of alternative splicing in these tissues. We separately traced the presence of the two forms (‘on’ and ‘off’) to generate an alternative splicing profile for each tissue. For each event, we determined the exon inclusion ratio from the expression levels of isoforms containing the 'on’ and the ‘off’ forms in that tissue: R = FPKM _on_/(FPKM _on_+FPKM _off_), and then compared the profiles to determine similarities and changes in splicing patterns among tissues. We used the relative inclusion ratio ^2^ to characterize such changes: ∆ = |Ri-Rj| between tissues *i* and *j*, and classified them based on the size of the difference. We separately trace exon skipping events that show large variation (‘switches’; ∆≥0.5), essentially switching between a minor-form and a major-form, and those that show milder variation. Note that all of these evaluations, based as they are on a single sample from each tissue, provide only a qualitative assessment of variation. Multiple replicates would be required to make any conclusions about the statistical significance of these changes between tissues.

**Figure 3.**
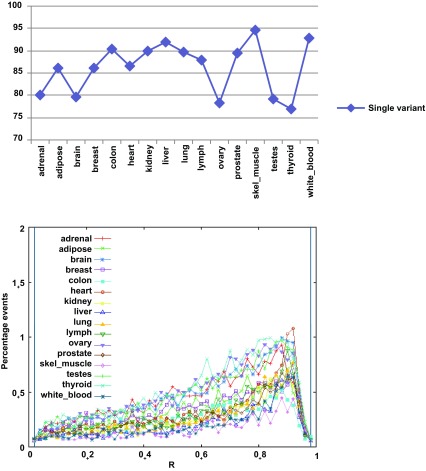
Splicing variation at skipped exon events as measured by the exon inclusion ratio R = FPKM _on_/(FPKM _on_+FPKM _off_) in the sixteen tissues. Most events within a given tissue are single variant (top). When both isoforms are present in a tissue, the exon is typically contained in the major form (R ≥ 0.5) (bottom).

**Figure 4.**
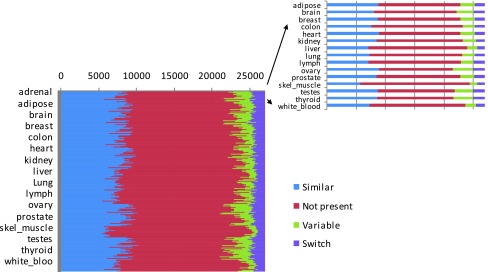
Splicing patterns for the 26,989 exon skipping events are compared between any two tissues, and events are classified by the difference in the splicing ratios. The 255 x 255 matrix shows the dynamics of exon skipping events between a tissue and each of the others. The numbers of similar (blue), variable (green), switch (purple) and not present (red) events between any two tissues are shown along one line.

**Figure S12.**
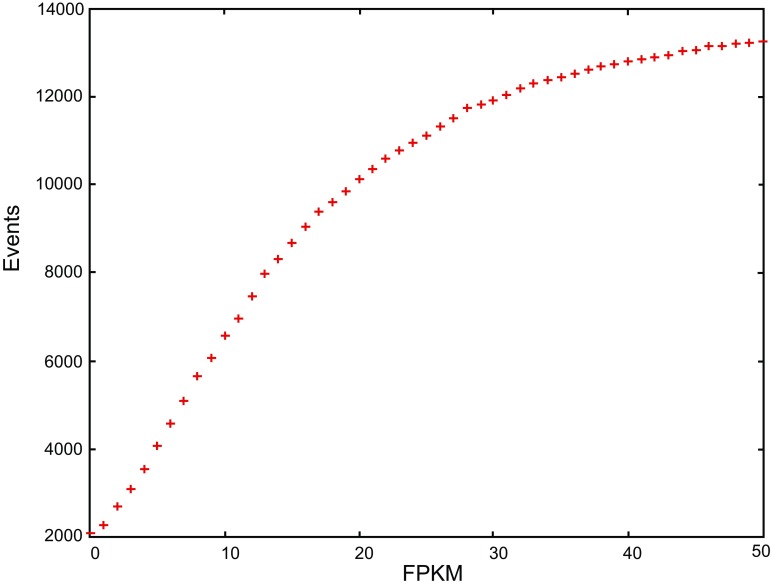
Expression levels of genes for the 13,946 events not comparable between adrenal and adipose tissues. 2,085 (15.0%) event genes are not expressed (FPKM<0.1), whereas 3,487 (33.7%) have FPKM values less than 10.0 and therefore may be incompletely reconstructed, which can cause a splice form to be missed.

Of the 26,989 exon skipping events, between 10,000–20,000 are present in any given tissue. Most events (77–95%) have only one of the forms expressed in a given tissue, and only 5–23% have both forms present in the same tissue ( Figure 3 and Supplemental Table S11). The exon-containing (‘on’) form is generally prevalent (R≥0.5). When comparing the profiles between two tissues ( Figure 4), 25–35% of the events show stable splicing patterns (∆<0.1), 5–10% are variable (0.1≤∆<0.5) and only 4–7% appear to switch. These proportions are quite similar among the tissues. Roughly 50–65% of the events are not comparable, with the event not found in either or both tissues. Further examination showed these to be due largely to the gene not being expressed (fragments per kilobase of transcript per million mapped (FPKM)<0.1) or harboring different splice forms, whereas we expect the number of incomparable events caused by computational artifacts to be very low. A significant portion of these genes were expressed at low-to-medium levels (FPKM≤10.0), which makes reconstruction difficult and may cause the event to be missed. (For an example, the comparison between the adrenal and adipose tissues is shown in Supplemental Figure S12). These observations suggest that both transcription and alternative splicing contribute significantly in shaping the transcriptomic differences among tissues, although more complete data sets and experiments are needed to be able to tease apart their specific contributions.

### Characterization of novel events

We contrasted known and newly found events to determine characteristics that could have made the latter difficult to discover with conventional (Sanger) data and methods, and to derive insights into the types of experiments that can help fill in the gaps in the alternative splicing catalog.

First, we analyzed the variability in splicing patterns of events, distinguishing between ‘switches’ and events exhibiting milder variation. There was a slight but statistically significant difference between the distributions of novel and known events (chi-square 274.7; p=0.0; Supplemental Table S13), with switches representing 69% of the known events and only 59% of the novel set.

**Figure 5.**
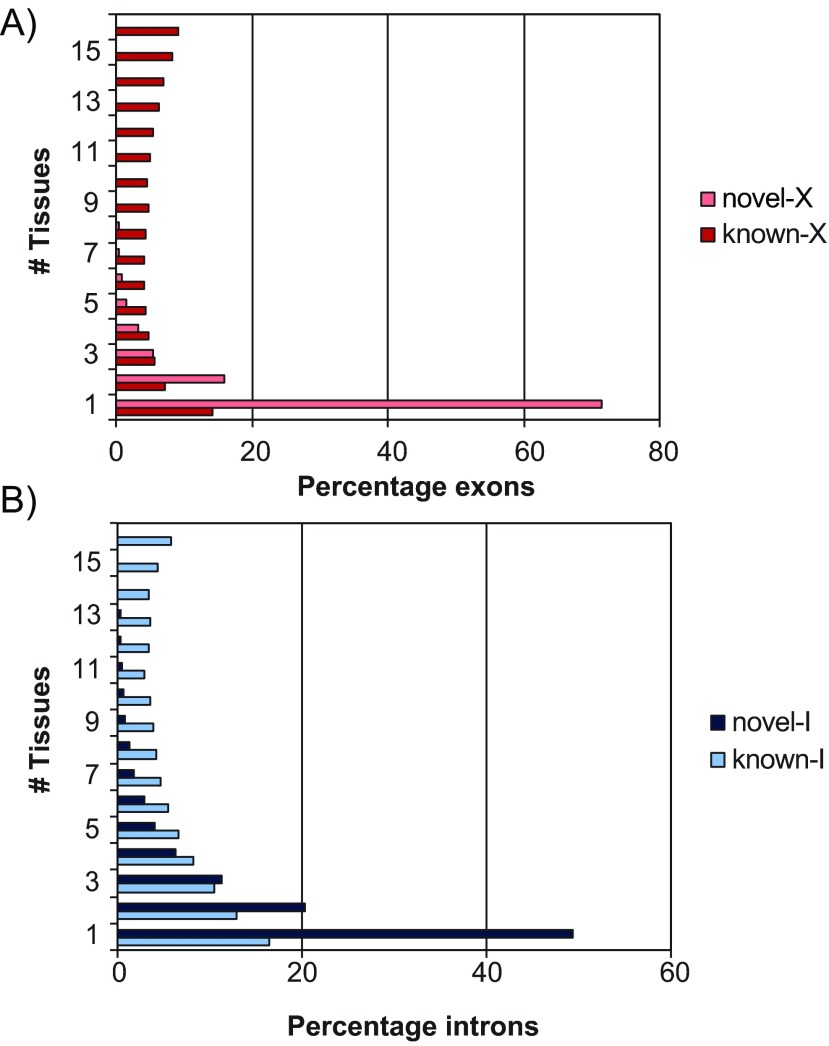
Distribution of novel and known features by the number of tissues in which they occurred. ( **A**) The percentage of exons found in 1, 2, …, 16 tissues are shown as horizontal bars, for the 2,914 novel exons (‘novel-X’) and 24,075 known exons (‘known-X’). ( **B**) Similarly for the 15,958 novel introns (‘novel-I’) and 11,031 known introns (‘known-I’).

Second, we assessed the tissue specificity of known and newly found exons and introns based on the data available ( Figure 5). For this test, we binned both the known and the novel features according to the number of tissues in which they were found. Not surprisingly, novel exons and introns were significantly more likely to appear in a small number of tissues compared to their known counterparts, but the prevalence was remarkable for exons. For instance, while novel introns were more likely to belong to a single tissue by a 3.0:1 margin (48% versus 16%), that margin for exons was 5:1 (71% versus 14%). Considering that our search turned out many more novel introns than exons, this observation suggests that targeted studies will be needed in the future to identify these highly tissue-specific exons.

## Discussion

Alternative splicing is a widely recognized RNA processing mechanism in eukaryotic species, playing a major role in the molecular biology of the cell, and within humans it has been implicated in multiple genetic disorders ^33^. The Human Genome Project created an initial map of splice variation more than a decade ago ^34, 35^. However, despite concerted efforts over the following years, this map is still inaccurate and incomplete. The Ensembl annotation ^15^, which is among the most complete to date, currently contains seven variants on average per protein coding gene. This is likely an underestimate, as more variants are added every day. The challenge of cataloging all alternative splice variants is daunting, considering that every tissue and cell type can have a different transcriptome, further differentiated by the condition of the cell at the time it was surveyed.

Unlike traditional methods that have mined heterogeneous cDNA sequences collected over time, RNA-seq experiments can survey the transcriptome of a cell type or tissue at great depth, allowing characterization of alternative splicing in much finer detail than previously. The main drawback to RNA-seq today is that its shorter reads are more challenging to assemble into long isoforms. To avoid some of the uncertainty associated with transcriptome assembly, we focused here on alternative splicing events within a transcript, each of which can be detected with a single read.

We found over 150,000 candidate alternative splicing events, including roughly 27,000 exon skipping events, most of which (65%) were novel. New introns (15,958) were the main source of novel events in our data set, but we also found a large number of new exons (2,914). A large majority of the new exons appear to be tissue-specific, with 71% present in only one tissue, which may explain why they have not been detected previously. Tissue-specific exons represent a clear and important contribution of alternative splicing to tissue differentiation, hence it is noteworthy that 2,085 (38%) of the 5,520 events in our data set were newly identified in this study. Both novel exons and novel introns were more likely to be tissue-specific than those already in the public annotation sets. This suggests that targeted experiments in different tissues or cellular conditions will be more productive in identifying novel splice forms in the future. This requirement is particularly relevant for identifying new exons, which have already been surveyed quite intensively, whereas even broad range RNA-seq experiments remain a rich source of new introns.

Our analysis of the 27,000 events across the sixteen tissues has also revealed insights into the dynamics of the alternative splicing repertoire and its role in tissue differentiation. With roughly 10–20% of the events showing variation across the tissues and 50–65% incomparable based on the existing data, the picture of alternative splicing contributions to tissue transcriptome differentiation vis-à-vis transcription is shaping up to be significant, albeit incomplete. Indeed, even in a deep and rich data set such as the Illumina Human Body Map, rare splice forms may be poorly represented or can be missed entirely. Also, our analyses here are based on a single experiment per tissue from a single individual, and therefore we cannot rule out polymorphic variation, although we expect its contribution to be small relative to tissue related differences ^2, 14^. Of course, experimentally testing the events ^36^ and replication on multiple biological samples, from different individuals, will be essential for full validation.

While there are ongoing efforts to incorporate alternative splicing information from RNA-seq data into gene annotation databases ^37^, there is yet no repository specifically for human alternative splicing events. Our analyses have identified thousands of putative alternative splicing events, which we have compiled into a catalog of exon skipping events derived from RNA-seq data from multiple human tissues. This collection will be a valuable resource for investigating the mechanisms and evolution of alternative splicing, and as a complement to existing annotation databases. Although this catalog adds substantially to the list of known alternative splicing events, many more RNA-seq experiments will be needed to fully characterize alternative splicing over the full spectrum of tissue types and cellular conditions. Our methods, as implemented in the ASprofile software, are freely available to allow others to create similar databases for other organisms or experimental systems.

## Materials and methods

### Sequence data

RNA-seq data for the Illumina Human Body Map Project were downloaded from http://www.ebi.ac.uk/arrayexpress/browse.html?keywords=E-MTAB-513&expandefo=on. For sequencing, samples for each of the 16 tissues (adrenal, adipose, brain, breast, colon, heart, kidney, liver, lung, lymph, ovary, prostate, skeletal muscle, testes, thyroid and white blood cells) were prepared by Illumina using their mRNA-Seq kit (Part #RS-100–0801). In brief, PolyA+ RNA was purified from 100 ng of total RNA with oligo-dT beads, and then fragmented with divalent cations under elevated temperature. First strand synthesis was performed with random hexamer and reverse transcriptase, and second strand synthesis with RNAseH and DNA PolI. Following cDNA synthesis, the double stranded products were end-repaired, a single "A" was added and then the Illumina PE adaptors were ligated on to the cDNA products. The ligation products were purified using gel electrophoresis. The target size range for these libraries was ~300 bp, such that the final library for sequencing would have cDNA inserts with sizes of ~200 bp long. One run of 2x50 bp paired-end sequencing was performed on the HiSeq2000 instrument, using one lane per tissue, to produce approximately 80 million read pairs per tissue (160 million sequences) ( http://www.ebi.ac.uk/arrayexpress/experiments/E-MTAB-513/protocols/).

### Reconstructing the tissues’ transcriptomes

To determine splice variants within each tissue, we aligned reads to the hg19 genome using TopHat v1.3.3 (parameters *‘-a 6 –F 0.05 –splice_mismatches=1 –max-multihits=10’*). To allow TopHat to detect as many splice junctions as possible, we provided an intron database extracted from the UCSC known Genes data set ( http://genome.ucsc.edu). Aligned reads were then assembled into transcript fragments using Cufflinks v0.9.3 (parameters *‘-F 0.05’*). We used Cuffcompare to compare these transfrags to the Ensembl v.61 annotation, and then Cuffdiff to redistribute reads along a high-confidence set of transcripts obtained after eliminating likely artifacts and assemblies not associated with Ensembl genes. Cuffcompare classifies assembled transcripts into multiple categories in relation to reference transcripts, including equal, contained, new splice isoform, intron-located, pre-mRNA fragment, repeat, etc. We retained only transcripts that were deemed ‘equal’, ‘contained’, or ‘new splice isoforms’ as part of our high-confidence set for each tissue. FPKM expression level values for this set were then re-estimated from the original alignments using Cuffdiff.

### Discovery of alternative splicing events

To determine alternative splicing events, we developed a software package, ASprofile, to analyze all pairs of transcripts in the sixteen tissues to determine exons included in one transcript and skipped in the other. We restricted the analysis to Ensembl genes with FPKM≥0.1, re-estimated by Cuffdiff as described above. We define an exon skipping event as a pair between an exon containing (‘on’) splice form and an exon skipping (‘off’) splice form, where the boundaries of the flanking introns are required to match precisely. To determine which events are novel, the exons and spanning introns were compared against several annotation data sets (CCDS, UCSC Genes, Ensembl v.61, H-ASDB and dbEST ^10^), allowing for a small difference (up to W=5 bp) at the endpoints. For comparison against ESTs, spliced alignments of all human dbEST sequences were produced with the program ESTmapper ^38^.

### Comparison of alternative splicing events across tissues

For each event, we calculated the exon inclusion ratio R = FPKM _on_/(FPKM _on_+FPKM _off_) for each tissue, similarly to Wang *et al.* ^2^, where FPKM _on_ is the combined FPKM of all isoforms containing the ‘on’ form, and similarly for FPKM _off_. To account for minor differences in the annotation of splice junctions, when calculating the expression level of an event we included contributions from splice forms in which the boundaries of the exon and flanking and spanning introns differed slightly (W≤10) from those of the annotated event. The relative inclusion ratio between two tissues, Δ _ij_ = | R _i_–R _j_ |, was determined for each event and used to classify events based on the size of the differences: stable (Δ < 0.1), variable (0.1 ≤ Δ < 0.5), ‘switch’ (Δ ≥ 0.5), or incomparable, when the event was not found in one or both tissues. For the tissue-specificity analysis, the largest difference between any two tissues was used to determine ‘switches’ versus ‘non-switches’.

### Implementation

**Figure S14.**
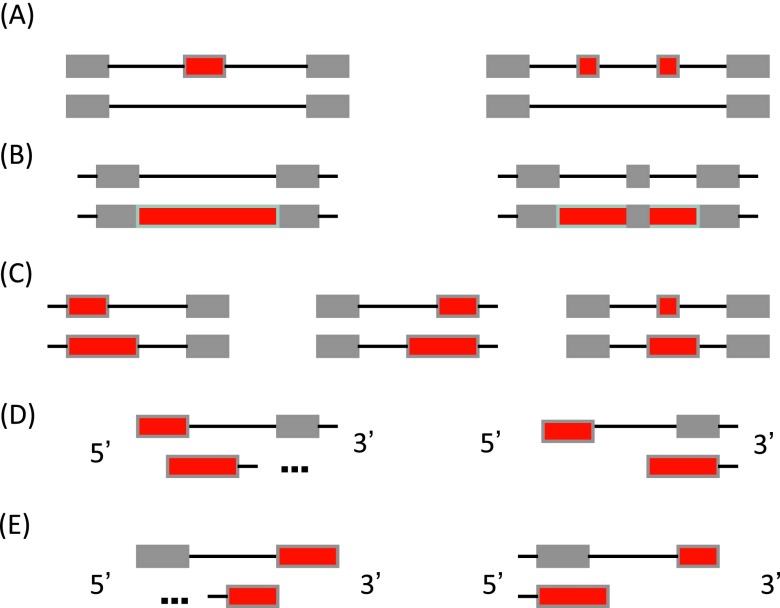
Classes of alternative splicing events detected by ASprofile by pairwise transcript comparisons. ( **A**) Exon skipping (SKIP) and cassette exons (MSKIP); ( **B**) retention of single (IR) and multiple (MIR) introns; ( **C**) alternative exon ends (AE); ( **D**) alternative transcription start site (TSS); and ( **E**) alternative transcription termination site (TTS). Alternatively spliced features are shown in red.

We implemented the methods in a software package, ASprofile, for discovering alternative splicing events in transcripts predicted from RNA-seq data and then comparing them across multiple conditions. ASprofile consists of programs for extracting ( *‘extract-as’*), quantifying ( *‘extract-as-fpkm’*) and comparing ( *‘collect-fpkm’*) alternative splicing events. *‘Extract-as’* takes as input a GTF transcript file, for instance one produced by a transcript assembly program or a set of gene annotations, and compares all pairs of transcripts within a gene to determine exon-intron structure differences that indicate an AS event. The following classes of events are currently implemented: exon skipping, cassette exons, alternative transcript start and termination, retention of single or multiple introns, and alternative exon ends ( Supplemental Figure S14). To determine alternative splicing events among multiple samples, a single input file must be created by concatenating the transcript files of individual samples, with the gene names *a priori* reconciled across the samples (for instance, by using the program Cuffcompare from the Cufflinks suite). The second program, *‘extract-as-fpkm’*, calculates the FPKM of each event from those of transcripts harboring the event in a given sample, allowing for small variations (up to V bp, where V is a user-specified value) at the boundaries of the exons and introns. Lastly, the script *‘collect-fpkm’* collects the FPKM event values for all RNA-seq samples, and calculates and compares splicing ratios across samples, which can be used to observe trends in the dynamics of alternative splicing profiles or to select promising candidates for laboratory testing. The software package is written in C and Perl and is available free of charge from the Zenodo repository ( http://zenodo.org/record/7068; doi:10.5281/zenodo.7068) and from our web site at http://ccb.jhu.edu/software/ASprofile.

## Figures and Tables

**Table S1.** Total number of RNA-seq reads from each of 16 human tissues. "Mapped" refers to the number of reads that had at least one and no more than ten alignments to the genome.

Tissue	Reads	Mapped	Properly paired
Adipose	154,600,144	138,872,338 (88.9%)	76,196,138 (49.3%)
Adrenal	148,945,742	133,883,290 (89.9%)	78,356,524 (52.6%)
Brain	147,026,094	134,791,012 (91.7%)	87,153,922 (59.3%)
Breast	151,724,430	135,987,146 (89.6%)	78,111,468 (51.5%)
Colon	164,874,886	150,966,150 (91.6%)	83,026,962 (50.4%)
Heart	165,837,568	155,841,749 (94.0%)	102,600,102 (61.9%)
Kidney	160,794,674	143,888,751 (89.5%)	84,285,000 (52.4%)
Liver	160,097,246	149,817,522 (93.6%)	94,840,760 (59.2%)
Lung	158,593,810	144,039,113 (90.8%)	90,728,136 (57.2%)
Lymph	164,156,314	146,044,267 (89.0%)	89,437,520 (54.5%)
Ovary	161,892,520	147,588,256 (91.2%)	81,207,460 (50.2%)
Prostate	164,668,152	150,477,990 (92.2%)	94,643,390 (57.5%)
Skeletal muscle	164,222,278	151,847,711 (91.6%)	95,036,702 (57.9%)
Testes	163,672,398	149,727,704 (91.5%)	83,729,866 (51.2%)
Thyroid	163,825,774	148,942,521 (90.9%)	80,513,364 (49.1%)
White blood cell	162,434,296	149,167,286 (91.8%)	87,806,618 (54.1%)
Total	2,557,366,326	2,333,252,527 (91.2%)	1,387,673,932 (54.3%)

**Table S2.** Performance of four transcript assembly programs in capturing exon skipping events from simulated RNA-seq data. Recall = TP/(TP+FN), Precision = TP/(TP+FP).

Exon skipping (ES)				
	Predicted	Correct	Recall	Precision
Cufflinks	632	561	**0.42**	**0.89**
IsoCEM	940	496	0.37	0.53
SLIDE	3022	311	0.23	0.10
Scripture	1724	1045	0.78	0.61

**Table S3.** Overview of results from the Cufflinks transcript assembly process. Cufflinks assembles short reads aligned to a genome into a set of transcript fragments (‘transfrags’ or ‘transcripts’ or ‘txpts’, below), grouped by locus (‘gene’).

Tissue	Genes*	Multi-exon	Txpts/gene ^†^	Transfrags	Single exon	Multiexon	Exons/txpt ^‡^
Adrenal	307,088	23,135	2.0	357,774	311,766	46,008	5.6
Adipose	134,348	21,793	1.5	158,142	126,207	31,935	6
Brain	239,971	24,435	1.8	218,818	238,497	43,321	6
Breast	191,620	23,553	1.6	222,525	185,869	36,656	5.5
Colon	116,723	23,503	1.3	133,953	103,071	30,882	5.1
Heart	149,951	21,537	1.5	177,059	144,829	32,230	6.5
Kidney	171,862	24,000	1.5	197,742	162,910	34,832	5.4
Liver	95,495	21,228	1.3	110,116	82,732	27,384	5
Lung	172,439	25,176	1.4	194,640	159,123	35,517	4.9
Lymph	188,709	23,766	1.5	216,692	179,938	36,754	4.9
Ovary	210,035	22,949	1.8	252,081	210,391	41,690	6.5
Prostate	161,526	24,584	1.4	186,159	150,732	35,427	5.5
Skel_muscle	95,684	19,695	1.2	105,209	82,359	22,850	4.7
Testes	220,459	29,546	1.6	259,043	212,894	46,169	5.9
Thyroid	187,169	23,357	1.8	224,647	184,751	39,896	6.3
White_blood	98,731	19,943	1.3	113,656	88,538	25,118	5.5

*Genes containing multi-exon transcripts.

^†^Averaged over multi-exon genes.

^‡^Averaged over multi-exon transcripts.

**Table S4.** Novel and known gene splice forms in the sixteen tissues, determined by comparison to the ENSEMBL v.61 annotation database using the program Cuffcompare. Cuffcompare compares each predicted transcript’s intron chain against those of the reference transcripts and classifies the transfrag as: *‘equal’* to a reference transcript, if their intron chains are identical; *‘contained’*, if included in a reference transcript’s; or as a *‘new splice form’*, if the intron chain has at least one splice junction in common with the reference transcripts (n.b., other Cuffcompare codes are not relevant and were omitted).

Set	Comparison to ENSEMBL v.61		
	Full-length (Equal)	Partial (Contained)	New splice forms
Adrenal	5,705	22,613	22,592
Adipose	5,593	22,293	11,233
Brain	6,497	20,673	19,329
Breast	5,665	24,190	13,665
Colon	4,689	28,715	9,310
Heart	6,103	17,614	11,701
Kidney	5,501	26,615	11,775
Liver	4,483	24,563	7,692
Lung	4,958	33,735	12,346
Lymph	4,574	33,682	14,364
Ovary	7,017	18,667	18,048
Prostate	5,673	27,183	12,470
Skel_muscle	3,318	33,999	4,999
Testes	7,077	23,648	17,306
Thyroid	6,487	21,098	16,667
White_blood	4,568	25,004	6,533

**Table S5.** Numbers of candidate alternative splicing events found in the Illumina Human Body Map data, by type.

Event type	Code	Count
Exon skipping (single exon)	SKIP	26,989
Exon skipping (multiple exons)	MSKIP	13,814
Alternative 5´ terminal exon*	TSS	97,399
Alternative 3´ terminal exon*	TTS	45,609
Intron retention (single intron)	IR	16,671
Intron retention (multiple introns)	MIR	2,636
Alternative exon boundaries	AE	22,767

*Includes 17,200 ‘default’ TSS (TTS) sites for 17,200 genes.

**Table S6.** Comparison of co-located ES events among the 26,989 events discovered from the Illumina Human Body Map data and the 25,114 exon skipping (ES) events extracted from the Ensembl v.61 annotations. *All* – all events co-located within *V* bp; Loci – clusters of co-located events (each with a representative ‘anchor’); *Var* ( *=All-Loci*) – variations (not including ‘anchors’), representing potential mapping artifacts.

V	Cufflinks (26,989 events)				Ensembl 61 (25,114 events)				% Diff	Potential artifacts
	All	Loci	Var.	%	All	Loci	Var.	%		
1	647	300	347	1.29	90	45	45	0.18	1.10	298
2	1271	585	686	2.54	143	71	72	0.29	2.26	608
3	2394	1153	1241	4.60	530	262	268	1.07	3.53	953
4	3079	1476	1603	5.94	724	359	365	1.45	4.49	1210
5	3507	1685	1822	6.75	839	412	427	1.70	5.05	1363
6	3803	1832	1971	7.30	894	440	454	1.81	5.49	1483
7	3923	1890	2033	7.53	930	456	474	1.89	5.65	1523
8	4003	1929	2074	7.68	959	471	488	1.94	5.74	1549
9	4126	1991	2135	7.91	1020	502	518	2.06	5.85	1578
10	4192	2057	2135	7.91	1068	500	568	2.26	5.65	1524

**Table S7.** Reproducibility of exon skipping (ES) discovery from transcripts assembled with different methods, and robustness with varying cutoffs (V bp) for the margin of error when comparing exon and intron boundaries.

Method	ES events	Found by other methods		
		V=0	V=5	V=10
Cufflinks	2,934	2,471	2,480	2,482
IsoCEM	1,486	1,361	1,364	1,365
SLIDE	1,418	860	884	895
Scripture	4,770	2,984	3,072	3,096

**Table S9.** Summary of novel features by comparison to four annotation databases (CCDS, UCSC Genes, Ensembl v. 61, and H-DBAS). Exon and intron boundaries were compared allowing for a small difference (up to V=0, 5 and 10 bp). Values in bold are those reported in the main text.

Novel feature	V=0	**V=5**	V=10
Exon+Intron	2,518	**1,430**	1,214
Exon only	1,954	**1,484**	1,368
Intron only	13,531	**14,528**	14,726
Known	8,986	**9,547**	9,681

**Table S11.** Exon skipping events expressed in each tissue sample, and number/percentage of these events that have only one expressed isoform in the sample.

Tissue	Events	Single variant
Adrenal	16,736	13,403 (80.1%)
Adipose	16,045	13,819 (86.1%)
Brain	17,415	13,865 (79.6%)
Breast	16,087	13,870 (86.2%)
Colon	13,802	12,464 (90.3%)
Heart	17,048	14,769 (86.6%)
Kidney	15,319	13,785 (90.0%)
Liver	12,390	11,388 (91.9%)
Lung	13,736	12,310 (89.6%)
Lymph	13,393	11,765 (87.8%)
Ovary	19,209	15,052 (78.4%)
Prostate	16,011	14,316 (89.4%)
Skel_muscle	10,160	9,614 (94.6%)
Testes	19,124	15,127 (79.1%)
Thyroid	18,689	14,362 (76.8%)
White_blood	12,954	12,017 (92.8%)

**Table S13.** Variability of novel exon skipping events compared to known events. Novel events are depleted in ‘switches’ compared to known events (test of homogeneity, df=2, chi-square 274.7, p = 0.0).

Events	Stable	Variable	Switches	Total
Novel	1,788	5,334	10,320	17,442
Known	688	2,241	6,618	9,547
Total	2,476	7,575	16,938	26,989

## Supplementary materials

### S2. Evaluation of transcript assemblers in detecting exon skipping (ES) events

To assess the feasibility of constructing an accurate repertoire of ES events, we evaluated the ability of Cufflinks and three other reference programs (Scripture ^29^, IsoCEM ^27^ and SLIDE ^28^) to capture ES events in a control data set. These four programs are representative for the classes of approaches currently employed by genome-guided transcript assembly methods. As a control data set, we simulated 200 million 75 bp paired-end reads using the program FluxSimulator ( http://flux.sammeth.net) starting from the GENCODE v17 gene annotations. We applied each program to the mapped reads, as described in the Methods for Cufflinks ( *‘-F 0.05’*) and using the defaults for all others, and detected ES events from the assembled transcripts with our ASprofile software. As gold reference, we used the set of events detected from the transcripts sampled by FluxSimulator, consisting of 1,327 exon skipping events.

As the comparison in Table S2 indicates, Cufflinks is the only tool that allows events to be detected with high precision, as needed to allow meaningful alternative splicing profile analyses. Notably, while Scripture is the most sensitive of the programs, its precision is low. As a caveat, these results reflect the performance of programs on simulated data, and may not be fully indicative of their behavior on real RNA-seq data.

### S6. Potential effects of imprecise splice junctions on the exon skipping (ES) estimates

One class of mapping artifacts that can contribute to creating false exon skipping events is that of ‘imprecise splice junctions’, where the same intron in different spliced reads is mapped by the alignment software at slightly different positions, typically within a few bp of each other, thus creating the appearance of several introns at that location. Such variations could lead to redundancies in reporting exon skipping events. To estimate the potential effects of imprecise splice junctions on our data set, we searched the 26,989 events against each other to identify those that are co-located and differ only slightly (by some maximum number of bases, *V* bp) in their exon-intron boundaries. (Note, however, that such events would also include all *true* alternative 5’ and 3’ exon ends). At each locus, we designate one event as ‘anchor’ (true), and refer to the co-located events as ‘variations’. We repeated the operation for 25,114 events discovered from the Ensembl v. 61 annotation with our software ASprofile, to create a baseline for comparison. For our data set of 26,989 events, labeled ‘Cufflinks’ in Figure S6 below, small ≤5 bp differences in splice junctions account for the majority of ES variations, beyond which their value as a fraction of the total number of ES events becomes relatively stable. In contrast, the percentage of Ensembl v. 61 event variations continues to increase roughly linearly with the distance from the ‘anchor’ splice junction. Comparing the two curves, we estimate that up to ~1,400 of ES events (5%) in our data set could be redundant (column 10 in the Table S6). We expect this value to be an overestimate, given that true small 5´ and 3´ exon alternative splicing variations are poorly represented in annotation databases.

### S7. Reproducibility of exon skipping event discovery with other assembly methods

To assess the reproducibility of our event discovery method, we compared the exon skipping events discovered from transcripts assembled with Cufflinks and with three other reference transcript assembly algorithms (Scripture ^29^, IsoCEM ^27^ and SLIDE ^28^). We applied each program to the adrenal sample of the Human Body Map data and used our program ASprofile to extract exon skipping events from the assembled transcripts.

As the results in Table S6 indicate, when compared to Cufflinks, IsoCEM and SLIDE find roughly half the number of events, whereas Scripture finds roughly 60% more events. (Allowing for small differences, up to V bp, in the coordinates of introns and exons to account for imprecise mapping of splice junctions, has negligible effects on the comparison results). There is generally good agreement among the methods, as most of the events predicted with one method can also be retrieved by at least one of the other methods. In particular, 84% (2,471 out of 2,934) of the events predicted when using Cufflinks can be confirmed by other methods. Additionally, our analysis reveals a number of potentially new events, most of them discovered by Scripture, which could be used to enrich our repository in the future. However, each program has its own characteristics and biases, and a careful analysis has to be performed before incorporating these events into the database.

## References

[1] Pan Q Shai O Lee LJ: Deep surveying of alternative splicing complexity in the human transcriptome by high-throughput sequencing. *Nat Genet.* 2008;40(12):1413–1415. 10.1038/ng.259 18978789

[2] Wang ET Sandberg R Luo S: Alternative isoform regulation in human tissue transcriptomes. *Nature.* 2008;456(7221):470–476. 10.1038/nature07509 18978772 PMC2593745

[3] Graveley BR: Alternative splicing: increasing diversity in the proteomic world. *Trends Genet.* 2001;17(2):100–107. 10.1016/S0168-9525(00)02176-4 11173120

[4] Stamm S Ben-Ari S Rafalska I: Function of alternative splicing. *Gene.* 2005;344:1–20. 10.1016/j.gene.2004.10.022 15656968

[5] Lorson CL Hahnen E Androphy EJ: A single nucleotide in the SMN gene regulates splicing and is responsible for spinal muscular atrophy. *Proc Natl Acad Sci U S A.* 1999;96(11):6307–6311. 10.1073/pnas.96.11.6307 10339583 PMC26877

[6] Narla G DiFeo A Yao S: Targeted inhibition of the KLF6 splice variant, KLF6 SV1, suppresses prostate cancer cell growth and spread. *Cancer Res.* 2005;65(13):5761–5768. 10.1158/0008-5472.CAN-05-0217 15994951

[7] Garcia-Blanco MA Baraniak AP Lasda EL: Alternative splicing in disease and therapy. *Nat Biotechnol.* 2004;22(5):535–546. 10.1038/nbt964 15122293

[8] David CJ Chen M Assanah M: HnRNP proteins controlled by c-Myc deregulate pyruvate kinase mRNA splicing in cancer. *Nature.* 2010;463(7279):364–368. 10.1038/nature08697 20010808 PMC2950088

[9] Hofstetter G Berger A Fiegl H: Alternative splicing of p53 and p73: the novel p53 splice variant p53delta is an independent prognostic marker in ovarian cancer. *Oncogene.* 2010;29(13):1997–2004. 10.1038/onc.2009.482 20101229

[10] Wheeler DL Barrett T Benson DA: Database resources of the National Center for Biotechnology Information. *Nucleic Acids Res.* 2008;36(Database issue):D13–21. 10.1093/nar/gkm1000 18045790 PMC2238880

[11] Gerhard DS Wagner L Feingold EA: The status, quality, and expansion of the NIH full-length cDNA project: the Mammalian Gene Collection (MGC). *Genome Res.* 2004;14(10B):2121–2127. 10.1101/gr.2596504 15489334 PMC528928

[12] Wang Z Gerstein M Snyder M: RNA-Seq: a revolutionary tool for transcriptomics. *Nat Rev Genet.* 2009;10(1):57–63. 10.1038/nrg2484 19015660 PMC2949280

[13] Mortazavi A Williams BA McCue K: Mapping and quantifying mammalian transcriptomes by RNA-Seq. *Nat Methods.* 2008;5(7):621–628. 10.1038/nmeth.1226 18516045 PMC13303166

[14] Gonzalez-Porta M Calvo M Sammeth M: Estimation of alternative splicing variability in human populations. *Genome Res.* 2012;22(3):528–538. 10.1101/gr.121947.111 22113879 PMC3290788

[15] Flicek P Amode MR Barrell D: Ensembl 2012. *Nucleic Acids Res.* 2012;40(Database issue):D84–90. 10.1093/nar/gkr991 22086963 PMC3245178

[16] Pruitt KD Harrow J Harte RA: The consensus coding sequence (CCDS) project: Identifying a common protein-coding gene set for the human and mouse genomes. *Genome Res.* 2009;19(7):1316–1323. 10.1101/gr.080531.108 19498102 PMC2704439

[17] Dreszer TR Karolchik D Zweig AS: The UCSC Genome Browser database: extensions and updates 2011. *Nucleic Acids Res.* 2012;40(Database issue):D918–923. 10.1093/nar/gkr1055 22086951 PMC3245018

[18] Takeda J Suzuki Y Sakate R: H-DBAS: human-transcriptome database for alternative splicing: update 2010. *Nucleic Acids Res.* 2010;38(Database issue):D86–90. 10.1093/nar/gkp984 19969536 PMC2808982

[19] Trapnell C Pachter L Salzberg SL: TopHat: discovering splice junctions with RNA-Seq. *Bioinformatics.* 2009;25(9):1105–1111. 10.1093/bioinformatics/btp120 19289445 PMC2672628

[20] Trapnell C Williams BA Pertea G: Transcript assembly and quantification by RNA-Seq reveals unannotated transcripts and isoform switching during cell differentiation. *Nat Biotechnol.* 2009;28(5):511–515. 10.1038/nbt.1621 20436464 PMC3146043

[21] Clark TA Schweitzer AC Chen TX: Discovery of tissue-specific exons using comprehensive human exon microarrays. *Genome Biol.* 2007;8(4):R64. 10.1186/gb-2007-8-4-r64 17456239 PMC1896007

[22] Xu Q Modrek B Lee C: Genome-wide detection of tissue-specific alternative splicing in the human transcriptome. *Nucleic Acids Res.* 2002;30(17):3754–3766. 10.1093/nar/gkf492 12202761 PMC137414

[23] de la Grange P Gratadou L Delord M: Splicing factor and exon profiling across human tissues. *Nucleic Acids Res.* 2010;38(9):2825–2838. 10.1093/nar/gkq008 20110256 PMC2875023

[24] Elliott DJ Grellscheid SN: Alternative RNA splicing regulation in the testis. *Reproduction.* 2006;132(6):811–819. 10.1530/REP-06-0147 17127741

[25] Li Q Lee JA Black DL: Neuronal regulation of alternative pre-mRNA splicing. *Nat Rev Neurosci.* 2007;8(11):819–831. 10.1038/nrn2237 17895907

[26] Sultan M Schulz MH Richard H: A global view of gene activity and alternative splicing by deep sequencing of the human transcriptome. *Science.* 2008;321(5891):956–960. 10.1126/science.1160342 18599741

[27] Li W Feng J Jiang T: IsoLasso: a LASSO regression approach to RNA-Seq based transcriptome assembly. *J Comput Biol.* 2011;18(11):1693–1707. 10.1089/cmb.2011.0171 21951053 PMC3216102

[28] Li JJ Jiang CR Brown JB: Sparse linear modeling of next-generation mRNA sequencing (RNA-Seq) data for isoform discovery and abundance estimation. *Proc Natl Acad Sci U S A.* 2011;108(50):19867–19872. 10.1073/pnas.1113972108 22135461 PMC3250192

[29] Guttman M Garber M Levin JZ: Ab initio reconstruction of cell type-specific transcriptomes in mouse reveals the conserved multi-exonic structure of lincRNAs. *Nat Biotechnol.* 2010;28(5):503–510. 10.1038/nbt.1633 20436462 PMC2868100

[30] van Dijk TB Gillemans N Stein C: Friend of Prmt1, a novel chromatin target of protein arginine methyltransferases. *Mol Cell Biol.* 2010;30(1):260–272. 10.1128/MCB.00645-09 19858291 PMC2798285

[31] van Dijk TB Gillemans N Pourfarzad F: Fetal globin expression is regulated by Friend of Prmt1. *Blood.* 2010;116(20):4349–4352. 10.1182/blood-2010-03-274399 20688955 PMC2993632

[32] McDaneld TG Hancock DL Moody DE: Altered mRNA abundance of ASB15 and four other genes in skeletal muscle following administration of beta-adrenergic receptor agonists. *Physiol Genomics.* 2004;16(2):275–283. 10.1152/physiolgenomics.00127.2003 14645738

[33] Singh RK Cooper TA: Pre-mRNA splicing in disease and therapeutics. *Trends Mol Med.* 2012;18(8):472–482. 10.1016/j.molmed.2012.06.006 22819011 PMC3411911

[34] Venter JC Adams MD Myers EW: The sequence of the human genome. *Science.* 2001;291(5507):1304–1351. 10.1126/science.1058040 11181995

[35] Lander ES Linton LM Birren B: Initial sequencing and analysis of the human genome. *Nature.* 2001;409(6822):860–921. 10.1038/35057062 11237011

[36] Richard H Schulz MH Sultan M: Prediction of alternative isoforms from exon expression levels in RNA-Seq experiments. *Nucleic Acids Res.* 2010;38(10):e112. 10.1093/nar/gkq041 20150413 PMC2879520

[37] Derrien T Johnson R Bussotti G: The GENCODE v7 catalog of human long noncoding RNAs: analysis of their gene structure, evolution, and expression. *Genome Res.* 2012;22(9):1775–1789. 10.1101/gr.132159.111 22955988 PMC3431493

[38] Florea L Di Francesco V Miller J: Gene and alternative splicing annotation with AIR. *Genome Res.* 2005;15(1):54–66. 10.1101/gr.2889405 15632090 PMC540277

