# Supplementary material for: Thousands of exon skipping events differentiate among splicing patterns in sixteen human tissues
Source: F1000Res. 2013 Nov 21;2:188. Originally published 2013 Sep 16. [Version 2] doi: 10.12688/f1000research.2-188.v2 (PMC3892928; doi:10.12688/f1000research.2-188.v2)
Supplement: Peer review history

# Peer review: Referee response for version 2

**DOI:** 10.5256/f1000research.3081.r2539

**Published:** 2014-04-22

**Authors:** Manuel Corpas

**Affiliations:** 1 The Genome Analysis Center, Norwich, UK

I am satisfied with the current revision. Thanks for the changes made.

I have read this submission. I believe that I have an appropriate level of expertise to confirm that it is of an acceptable scientific standard.

# Peer review: Referee response for version 2

**DOI:** 10.5256/f1000research.3081.r2538

**Published:** 2014-03-10

**Authors:** Peter Robinson

**Affiliations:** 1 Institute for Medical Genetics, Universitätsklinikum Charité, Berlin, Germany

Thanks for adding table S2. This is a useful and interesting article, I have no further suggestions.

I have read this submission. I believe that I have an appropriate level of expertise to confirm that it is of an acceptable scientific standard.

# Peer review: Referee response for version 1

**DOI:** 10.5256/f1000research.2196.r2236

**Published:** 2013-10-29

**Authors:** Peter Robinson

**Affiliations:** 1 Institute for Medical Genetics, Universitätsklinikum Charité, Berlin, Germany

In this article, the authors address the question of how much alternative splicing can be identified in 16 different human tissues, and how much of the alternative splicing is specific for one or another tissue. To do so, they analyzed 50-bp paired-end sequences from 16 different tissue samples sequenced relatively deeply (ca. 80 million read pairs per tissue sample). The data has been made available by Illumina as the Human Body Map project. Computational analysis was then performed with the programs TopHat and Cufflinks. This type of analysis assembles the reads into a set of transcripts that are compatible with the splicing patterns inferred from reads that are split over multiple exons.  Between 23,000–46,000 partial or complete transcript assemblies (“transfrags”) were obtained per tissue. Approximately 5,000–20,000 transfrags per tissue, or 11–45% of the total, were identified as potentially novel splice forms. As the authors note, it is still difficult to assemble complete transcripts from relatively short RNA-seq reads in this way, and they thus make the very reasonable decision to concentrate their further analysis on alternative splicing events that are likely to be valid. Thus, it is easier to analyze the spectrum of exon skipping/inclusion events at a particular exon.

In general, the article is well done and will be of interest to those involved with RNA-seq or alternative splicing. One particularly interesting, if not entirely surprising, finding of this study is the fact that the majority of the novel exon insertion events showed a high degree of tissue specificity, thus providing an obvious explanation of why the corresponding exons are not yet in public databases. This suggests that efforts should be spent to characterize the full range of splicing in tissues in order to understand their biology.

Comments:

1. It would be useful if the authors could provide more discussion of the current state of the art of transcript identification using RNA-seq reads. In particular, the authors discuss three other transcript assembly programs in the supplement (S6), finding that they identify 50-60% of the isoforms called by Cufflinks. Have the authors performed their downstream analysis using the results of one of these programs? How different would the results be?
2. The authors should try to place their results in the context of many other efforts at identifying novel isoforms from the literature and discuss the relative merits of their approach.
3. It would be useful to have a table with quality metrics for this dataset, e.g., Phred scores, percent of mapable reads etc. - table S1 could be extended for this purpose.
4. Although much of the results deal with exon skipping/insertion events, it would be interesting to hear a little about results for other classes of alternative splicing that can be identified by the authors’ software, e.g. alternative transcription start and termination sites.
5. In the results section, it would help the presentation of the material to provide a brief description of the relative inclusion ratio at this portion of the text and to define what i and j are (i.e., take some of the material from the methods section that explains this).

I have read this submission. I believe that I have an appropriate level of expertise to confirm that it is of an acceptable scientific standard.

# Response

**Published:** 2013-11-18

**Authors:** Liliana Florea

**Affiliations:** Johns Hopkins University, USA

We thank Peter Robinson for his insightful comments and suggestions, which we address in the following:

- Transcriptome assembly represents a complex topic on its own, and is only a tangent to our work, therefore we believe that it would be best addressed in detail elsewhere. We wish to point the reviewer and the readers to a review on current transcript assembly methods that we wrote recently, to appear in IEEE/ACM Transactions in Bioinformatics and Computational Biology journal. We also included a brief evaluation of several transcript assemblers on a simulated data set (new Table S2), showing that Cufflinks is the only program among those tested that can identify alternative splicing events with high enough accuracy (~90%) to allow meaningful downstream analyses, though we will explore combinations of these programs in the future. Minor correction to the statistic quoted by the reviewer: 84% of the Cufflinks-predicted exon skipping events are reproducible by other methods. 
- There is indeed a rich body of work in the area of alternative splicing. Notably, two early studies (references 1 and 2) highlighted the extent of alternative splicing in the human genome based on analyses of RNA-seq data, albeit those data sets were not nearly large enough to be able to characterize it in detail, and a more recent study (Merkin *et al*., 2012) extended those analyses in the context of multiple species. Similarly, there are now several methods (e.g., MISO, SpliceTrap and MATS) that infer or quantify exon skipping events directly from RNA-seq reads, and therefore are complementary to our approach. Using a very large data set, our work provides a more detailed picture of alternative splicing variation and produces both an easy-to-use tool and a database that we believe will be valuable in studies of alternative splicing mechanism and function.
- We added percentage of mapped reads and number of properly paired reads to Table S1, as indicators of read alignment quality.
- We think that analyzing other types of events is indeed a very interesting experiment, which due to scale we plan to address in our future work.
- The definitions were already in the main text (page 7, section “Characterization of exon skipping events”), but we have clarified i and j.

# Peer review: Referee response for version 1

**DOI:** 10.5256/f1000research.2196.r1828

**Published:** 2013-10-07

**Authors:** Manuel Corpas

**Affiliations:** 1 The Genome Analysis Center, Norwich, UK

The article 'Thousands of exon skipping events differentiate among splicing patterns in sixteen human tissues' presents a systematic analysis of alternative splicing of the Illumina Body Map RNA-seq set, probably the most complete set of RNA-seq data to date. This data set consists of 160M reads each for 16 tissues, each from a different individual. 

From the standpoint of a reviewer I consider this article to be of very good quality, and have very much enjoyed reviewing it. There are a few suggestions I hope could help improve this work.

1. P3. pp.2: Has alternative splicing profiling been attempted before? If so, could this please be shown in the background section? If not, how does this technique specifically differ from what has been done before? Some more context would be appreciated.
2. P3. pp.3: What is the effect of comparing tissues from 16 different individuals? I would expect that the genetic variation of each individual would have some effects in the actual AS patterns observed when compared. I see that these samples belong to people with different ethnic backgrounds and ages. I would be interested to know what the authors think about how different results would be if all 16 tissues had been from one individual.
3. I tried to compile ASprofile on a mac and I got this error:
  libc.h:15:20: error: malloc.h: No such file or directory
  I solved this by changing this line with #include <stdlib.h>. Perhaps you might want to add these lines, so that mac users do not encounter the problem:
  #if !defined(__APPLE__)
  #include <malloc.h>
  #endif
4. Although the license for ASprofile is available in the Zenodo page, it might also be a good idea to mention it somewhere in the article. Not mentioning a license may make some users uneasy, even if it says it is open source.
5. In S1, how many of the mapped reads were properly paired?
6. Why did you use such old versions of Ensembl genes (Ensembl 61, February 2011), UCSC Genes, CCDS genes and H-DBAs? I also find that the TopHat (v1.3.3) and Cufflinks (v0.9.3) versions are quite old. The current version for TopHat is 2.0.9 and the current version for Cufflinks is 2.1.1. The current version of Ensembl is version 73, September 2013. The usage of such old versions concerns me a little, particularly those of the gene annotations, given that a lot of the results that are presented in this work rely heavily on the comparison of AS profiles against those annotations. Some of the results might be different if a newer version of the annotations were up-to-date.
7. P5. pp.5: The example of a novel event in CHTOP does not provide clear evidence as to how many reads support the novel exon. Please provide some numbers.
8. P7. pp1: Please could you provide evidence for the novel putative intron retention event located in the chr7:123269489-123270019 region?
9. In Figure S7.B, I would appreciate some text describing the biological significance of having  the 100 bin in the x-axis with the greatest bars. Overall I am unable to understand what this figure means. 

I have read this submission. I believe that I have an appropriate level of expertise to confirm that it is of an acceptable scientific standard.

# Response

**Published:** 2013-11-18

**Authors:** Liliana Florea

**Affiliations:** Johns Hopkins University, USA

We wish to thank Manuel Corpas for his thorough review and suggestions. In responding to his comments:

- We would like to point to our answer to question 2 from Reviewer 1, where we more broadly address this and other related questions. 
- An earlier study by Wang *et al.* (reference 2) also tackled polymorphic splicing differences, finding that these represent a much smaller fraction of the AS variation compared to tissue differences. Therefore, while some polymorphic differences exist, they are not likely to distort the picture we paint in this study. We agree that analyzing data from multiple tissues in the same individual would be highly informative and we are actively looking for suitable data sets, which are not available at the moment.
- We thank the reviewer for taking the time to test the software. We updated the code and included a note on the license (GNU GPL) in the text (page 3).
- We have included proper read pairing information for all tissues in Table S1.
- With respect to the versions of the software, we note that at the time we ran our analyses we used the latest versions of each of these programs and databases, however our paper was caught up in the reviewing process at another journal for over a year. After two rounds of very slow reviews and multiple requests for revisions, the other journal was still not satisfied and therefore we decided to submit to F1000Research, which has far faster publication turnaround. We should emphasize that none of the previous reviewers’ comments questioned the validity of our results, and our revisions did not substantively change any of our conclusions. Nevertheless, we also searched the ‘novel’ events against the aggregate set of Ensembl 73, GENCODE v.17 and RefSeq representing the most recent versions of these collections, and only found 21 exons and 9 introns (note that these did not include the events depicted in Figures 2 and S9). Hence, the results discussed in the manuscript still hold.
- We have added read support information for the exon skipping event in Figure 2, and for the putative intron retention event in Figure S9.
-  The ‘100’ bin in Figure S7 (now S8) shows the proportion of introns supported by more than 100 reads, for each of the three types of introns. Most events have deep support (>=100 reads) for the flanking introns, and to a lesser extent for the spanning intron, which is consistent with our earlier finding that the exon tends to be skipped in the minor form (Figure 3).
